# Electrophysiological Mechanisms of Bayés Syndrome: Insights from Clinical and Mouse Studies

**DOI:** 10.3389/fphys.2016.00188

**Published:** 2016-05-31

**Authors:** Gary Tse, Eric Tsz Him Lai, Jie Ming Yeo, Bryan P. Yan

**Affiliations:** ^1^Li Ka Shing Faculty of Medicine, School of Biomedical Sciences, University of Hong KongHong Kong, China; ^2^School of Medicine, Imperial College LondonLondon, UK; ^3^Department of Medicine and Therapeutics, The Chinese University of Hong KongHong Kong, China; ^4^Department of Epidemiology and Preventive Medicine, Monash UniversityMelbourne, VIC, Australia

**Keywords:** Bayés syndrome, inter-atrial block, intra-atrial block, conduction, electrophysiological remodeling, structural remodeling

## Abstract

Bayés syndrome is an under-recognized clinical condition characterized by inter-atrial block (IAB). This is defined electrocardiographically as P-wave duration > 120 ms and can be categorized into first, second and third degree IAB. It can be caused by inflammatory conditions such as systemic sclerosis and rheumatoid arthritis, abnormal protein deposition in cardiac amyloidosis, or neoplastic processes invading the inter-atrial conduction system, such as primary cardiac lymphoma. It may arise transiently during volume overload, autonomic dysfunction or electrolyte disturbances from vomiting. In other patients without an obvious cause, the predisposing factors are diabetes mellitus, hypertensive heart disease, and hypercholesterolemia. IAB has a strong association with atrial arrhythmogenesis, left atrial enlargement (LAE), and electro-mechanical discordance, increasing the risk of cerebrovascular accidents as well as myocardial and mesenteric ischemia. The aim of this review article is to synthesize experimental evidence on the pathogenesis of IAB and its underlying molecular mechanisms. Current medical therapies include anti-fibrotic, anti-arrhythmic and anti-coagulation agents, whereas interventional options include atrial resynchronization therapy by single or multisite pacing. Future studies will be needed to elucidate the significance of the link between IAB and atrial tachyarrhythmias in patients with different underlying etiologies and optimize the management options in these populations.

## Introduction

The first case of inter-atrial block (IAB) was first described by Bachmann (1941), who recognized the significance of P-wave splitting on the ECG, some 25 years after he described the anatomy of Bachmann's bundle (Bachmann, 1916). Dr Bayés de Luna was the first who provided a clear description of atrial conduction block in 1979, classifying them into either inter- and intra-atrial (Bayés de Luna, 1979). In recognition of his numerous contributions to the understanding of IAB (Bayes de Luna et al., 1985), this disease was later named Bayés syndrome (Conde and Baranchuk, 2014).

**Figure 1:**
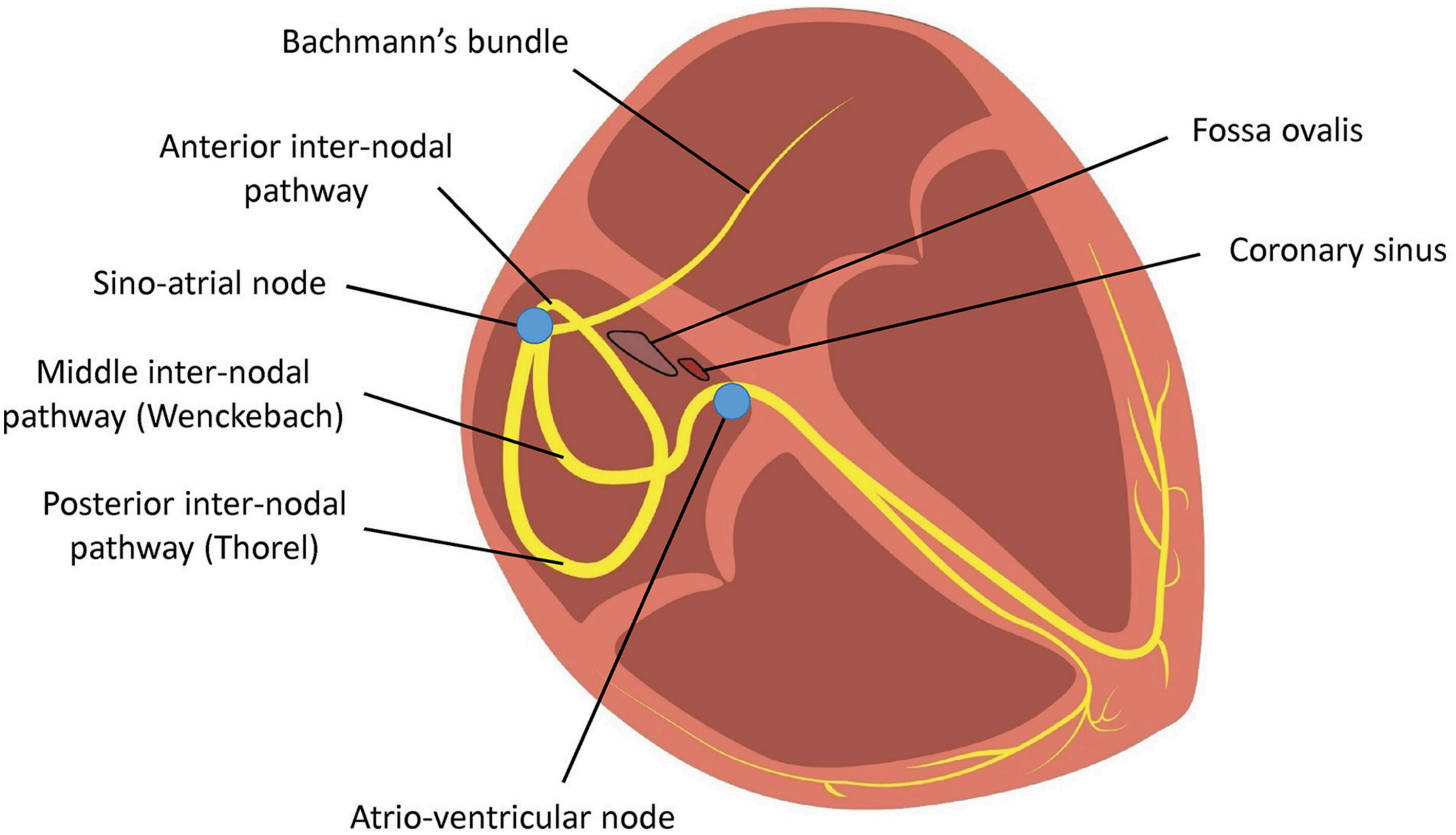
**Conduction of action potentials from the sinoatrial node across the right atrium to the atrioventricular node occurs via three inter-nodal tracts**. Conduction from the right atrium to the left atrium occurs via Bachmann's bundle.

The cardiac conduction system starts at the sinoatrial node, which is the pacemaker responsible for initiating action potentials (APs) that are conducted through the right atrium via three distinct inter-nodal pathways to the atrioventricular node. These are the anterior, middle (Wenckebach) and posterior (Thorel) pathways (Figure 1; Conde et al., 2015). Inter-atrial conduction from the right to left atrium occurs most frequently along Bachmann's bundle, which branches from the anterior internodal pathway (James, 1963; Racker, 1989), but it can also pass through the coronary sinus or the fossa ovalis (Tapanainen et al., 2009).

**Figure 2:**
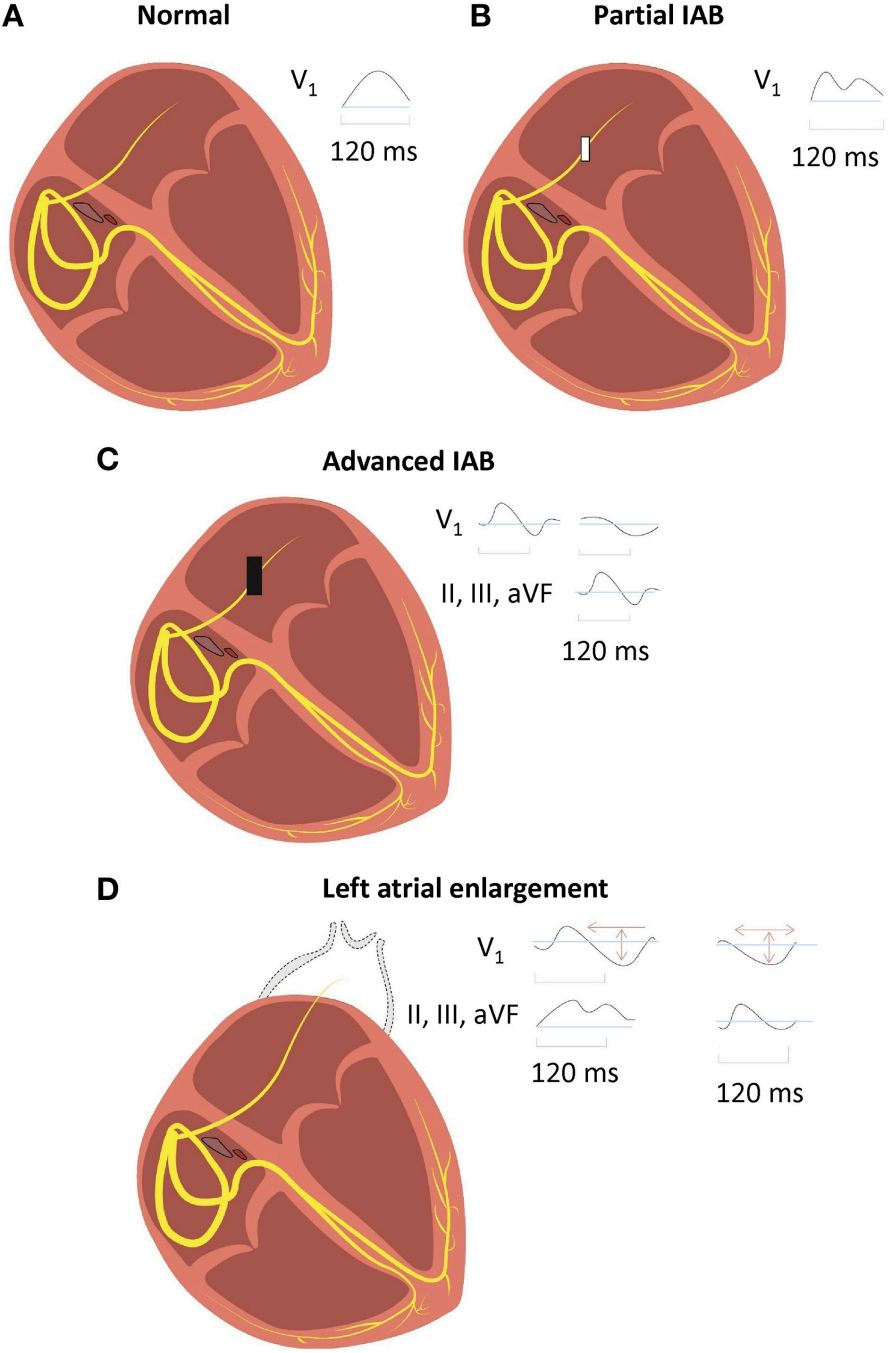
**Normal inter-atrial conduction (A), partial (B), and advanced (C) inter-atrial block (IAB), with distinct electrocardiographic findings**. IAB should be distinguished from left atrial enlargement (LAE) **(D)**, where there is conduction delay from lengthening of Bachmann's bundle in the absence of block.

IAB is caused by impaired conduction along Bachmann's bundle. Two definitions of IAB have been proposed. The classification adopted in the consensus report divides it into either partial (P wave duration > 120 ms) or advanced (P wave duration > 120 ms with biphasic morphology in the inferior leads; Bayés de Luna, 1979). Alternatively, similar to sinoatrial or atrioventricular block, it can be divided into first (partial), second (first degree with intermittent conduction through Bachmann's bundle) or third degree (advanced; Figure 2; Bayes de Luna et al., 2012; Chhabra et al., 2014). Partial or first degree IAB is characterized by prolonged P wave duration of >120 ms, with bifid (notching) with dome-and-spike morphology on the ECG. The definition of a normal P-wave duration is a contentious issue because 120 ms has been considered by some clinicians to be normal, yet the upper limit was defined by the World Health Organization and the International Society and Federation of Cardiology Task Force to be 110 ms (Willems et al., 1985). This may arise from difficulty in accurately measuring the P-wave duration (Baranchuk et al., 2016), which is in part due to baseline noise (Magnani et al., 2010). Intra- and inter-observer variability also contributes to inconsistent reported values (Dilaveris et al., 1999). Nevertheless, for first degree or partial IAB, a recent consensus report agreed on the definition of P-wave duration > 120 ms (Bayes de Luna et al., 2012). This is in keeping with a previous study demonstrating the modal P-wave duration to be 120 ms in first-degree IAB (Ariyarajah et al., 2006b).

In second degree IAB, the P-wave shows an initial invariant component but a second component with varying morphology within the same ECG. This is commonly seen in atrial aberrancy (Chung, 1970), with concealed atrial conduction from post-ectopic inhibition (Chung's phenomenon; Chung and Chung, 1972). Bachmann's bundle has a long effective refractory period (ERP; Vollmann et al., 2005), during which Na^+^ channels cannot be reactivated. Therefore, a premature AP will not be able to pass through this bundle, but has to take another and potentially longer path. Second degree IAB can occur in an absence of a premature atrial beat. A limitation of the definition of second degree IAB is that to diagnose intermittent conduction through Bachmann's bundle, a constant interval between two P-waves is needed. If this interval is not constant, then it is possible that variable morphologies can be explained by APs with different initiation sites, for example, opposite sides of the crista terminalis.

In advanced or third degree IAB, there is a biphasic P-wave in the inferior leads with a negative terminal deflection below the isoelectric line. As conduction via Bachmann's bundle is completely blocked, the AP wave must pass through another pathway, such as the coronary sinus. This may result in retrograde activation of the left atrium in the caudo-cranial direction, producing a negative terminal deflection (Bayes de Luna et al., 1988; Cosio et al., 2004). However, if the wave passes through the rim of the fossa ovalis, then retrograde activation may not necessarily occur.

Advanced IAB is clinically important as there is a high chance of developing supraventricular tachycardia if it is left untreated (Bayes de Luna et al., 1999). Left atrial enlargement (LAE) is often associated with, but should be distinguished from, IAB (Mehrzad and Spodick, 2014). LAE leads to prolonged inter-atrial conduction times because of increased stretch and lengthening of Bachmann's bundle (Boineau, 2005). This delay is due to increased distance of conduction rather than block in the bundle *per se*. In the inferior leads, there are biphasic P-waves but without the terminal negative deflections seen in third degree IAB. LAE can be diagnosed on the ECG by a biphasic P-wave on V1 together with an area under the curve for the second phase < 40 mm.ms (Chhabra et al., 2014).

**Figure 3:**
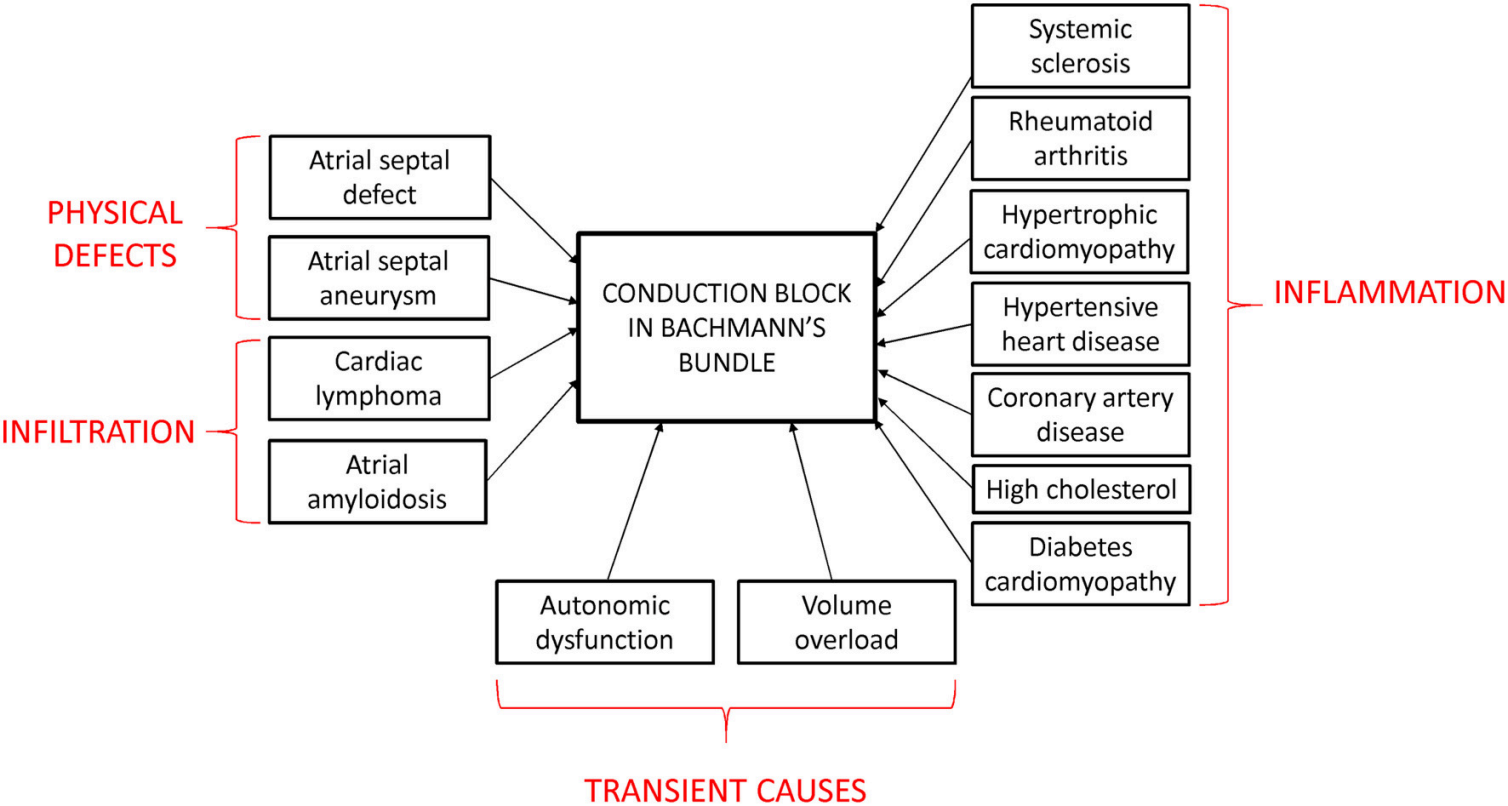
**Diseases leading to IAB**.

The prevalence of IAB is age-dependent, increasing from 5.4% at < 20 years old to 60% at >50 (Jairath and Spodick, 2001; Asad and Spodick, 2003; Ariyarajah et al., 2005; Gialafos et al., 2007; Martinez-Selles et al., 2016b). This is likely the result of aging-related fibrosis, which would result in impaired AP conduction through the atria (Gramley et al., 2009; Fleg and Strait, 2012). The risk factors for developing IAB are coronary artery disease, hypertension, diabetes mellitus and hypercholesterolemia (Figure 3; Ariyarajah et al., 2006a). IAB can be caused by structural defects of the conducting pathway, such as atrial septal defect (Thilen et al., 2007) or aneurysm (Okutucu et al., 2010), or infiltration of the bundle from cardiac lymphoma (Engelen et al., 2005; Peyrou et al., 2013) or amyloidosis (Rocken et al., 2002). Alternatively, inflammation can induce cardiac structural remodeling, which can occur in hypertrophic cardiomyopathy (Szili-Torok et al., 2014) or systemic inflammatory diseases such as scleroderma and rheumatoid arthritis (Mizuno et al., 1999; Acar et al., 2009). Transient IAB may be related to autonomic dysfunction, increased atrial strain or electrolyte abnormalities. For example, it was observed in decompensated heart failure with increased atrial strain from volume overload, which disappeared after its resolution using diuretic therapy (Song et al., 2002; Proietti et al., 2012), or hemodialysis patients with vomiting (Enriquez et al., 2015). In terms of disease progression, it takes around 66 months to progress from a normal P-wave duration to advanced IAB (Ariyarajah et al., 2007b). IAB is important because of increased risks of atrial arrhythmias (tachycardia, flutter, fibrillation) complicated by LAE and electro-mechanical discordance (Ariyarajah et al., 2007a). This predisposes to increased thrombosis in obstructive sleep apnea (Can et al., 2009; Cagirci et al., 2011; Maeno et al., 2013) and to myocardial ischemia (Myrianthefs et al., 1991), cerebral vascular accidents (Lorbar et al., 2005), and mesenteric ischemia (Chhabra et al., 2012).

IAB can be managed with pharmacological therapy, such as angiotensin-converting enzyme (ACE) inhibitors, which can delay the progression from first degree to third degree IAB (Ariyarajah et al., 2007b). It can also be managed by interventional procedures, such as multi-site or single site pacing at the triangle of Koch or Bachmann's bundle. It is unclear whether there is any benefit in early treatment. Atrial resynchronization therapy can be used to correct for left-sided atrioventricular dyssynchrony arising from IAB (Daubert et al., 2004).

## Electrophysiological mechanisms of Bayés syndrome

To understand the electrophysiological mechanisms of IAB and how it increases atrial arrhythmic risk, the determinants of AP conduction through the myocardium must first be considered (Kléber and Rudy, 2004; Tse and Yeo, 2015; Tse et al., 2016b). This can be described by the core conduction theory (Barr et al., 2003). Conduction velocity (CV) depends on both passive and active electrical properties. Passive properties refer to the resistive and capacitive components and the architecture of the myocardium. They include the axial resistance (r_i_) of both the myoplasm (Thomas et al., 2003) and the gap junctions between cardiomyocytes (Rohr et al., 1998; Chen et al., 2007), resistance of the extracellular space (r_o_), and the membrane capacitance (C_m_). Active membrane properties refer to the voltage-gated ionic conductances: the most important conductance is that of Na^+^ channels, which mediates *I*_Na_ and determine the AP upstroke. Reduced CV can arise from increased r_i_, r_o_ or C_m_, decreased maximum upstroke velocity (dV/dt_max_, from reduced Na^+^ current density, Nattel, 2008; Tse et al., 2016g) or decreased myocardial excitability given by 1/(Threshold Potential–Resting Membrane Potential). Cardiac fibrosis can decrease CV by the following mechanisms: reduced cardiomyocyte-cardiomyocyte coupling, which increases r_i_, or increased fibroblast-cardiomyocyte coupling, which increases C_m_ (Tse and Yeo, 2015). Moreover, fibroblast-cardiomyocyte coupling can depolarize cardiomyocytes (Rohr, 2012; Kohl and Gourdie, 2014; Thomsen and Calloe, 2016), leading to Na^+^ channel inactivation and reduced dV/dt_max_. It is increasingly recognized that passive and active properties are not independent of each other, since gap junctions and Na^+^ channels co-localize in the connexome, and their close proximity to each other could enable ephaptic conduction (Rhett and Gourdie, 2012; Veeraraghavan et al., 2012, 2014a,b,c, 2015; Rhett et al., 2013; George et al., 2015). Increased risk of arrhythmogenesis by either circus-type or spiral wave reentry can be explained by a reduction in excitation wavelength (λ) given by CV × ERP (Wiener and Rosenblueth, 1946; Smeets et al., 1986; Vaidya et al., 1999; Tse, 2015; Tse et al., 2016f, in press). Selective atrial fibrosis could increase the heterogeneity of conduction by allowing micro-reentry to take place in smaller areas in atrial fibrosis (Spach and Josephson, 1994; Verheule et al., 2004).

### Inter-atrial block can arise from abnormal function or expression of sodium channels and gap junctions

**Figure 4:**
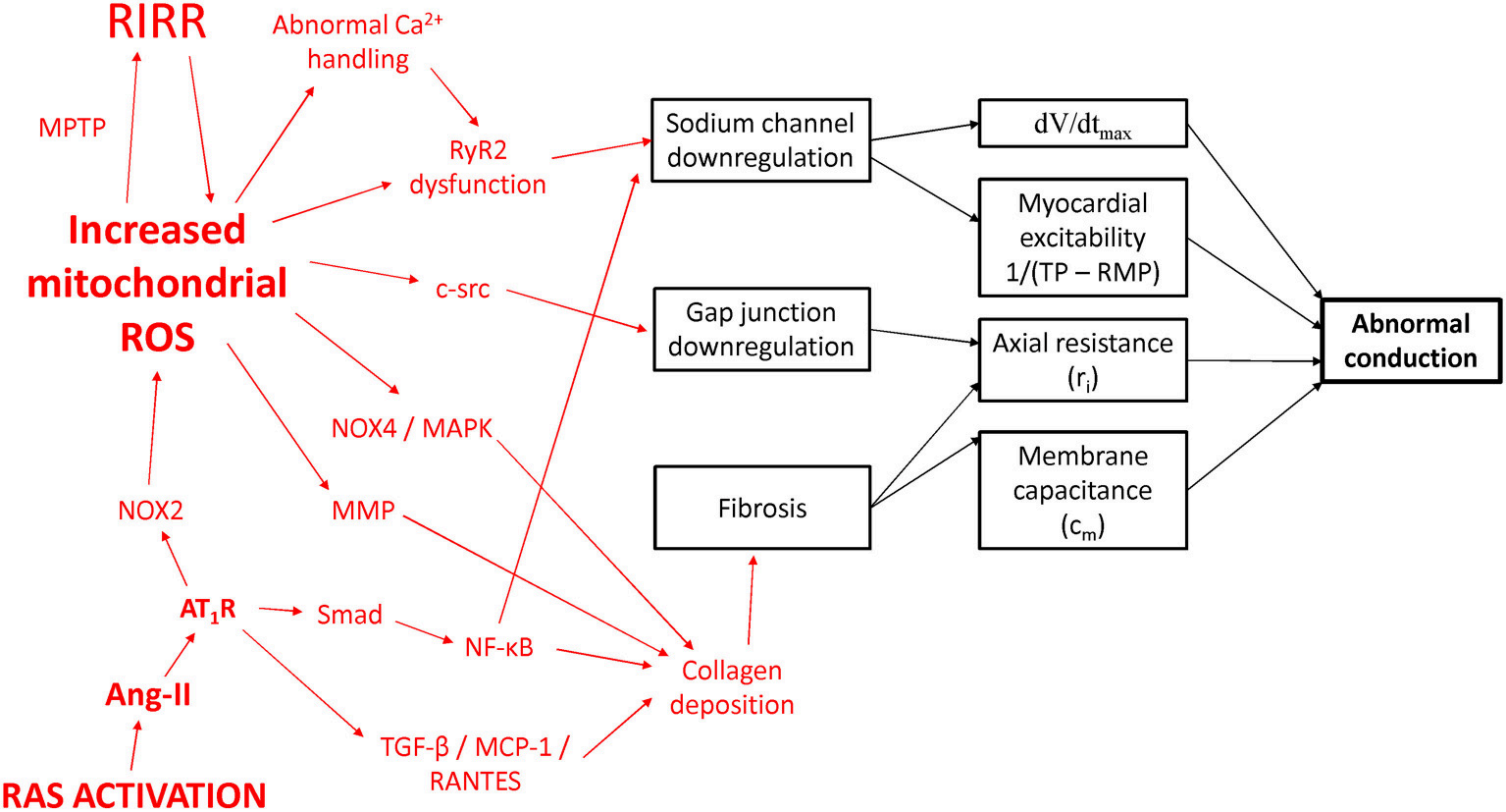
**Potential molecular mechanisms leading to conduction abnormalities in IAB**. RAS, renin-angiotensin system; Ang-II, angiotensin II; AT1R, angiotensin II receptor isoform 1; NOX, NADPH oxidase; MAPK, mitogen-activated protein kinase; MCP-1, monocyte chemoattractant protein-1; MMP, matrix metalloproteinase; MPTP, mitochondrial permeability transition pore; NF-κB, nuclear factor kappa-light-chain-enhancer of activated B cells; RANTES, Regulated on Activation, Normal T Cell Expressed and Secreted; ROS, reactive oxygen species; RIRR, ROS-induced ROS release; TGF-β, transforming growth factor-beta; RyR2, ryanodine receptor isoform 2.

The conditions predisposing or causing IAB can affect any of these parameters. Animals have been extensively used to study cardiac arrhythmogenesis in a number of clinical conditions because of their amenability to genetic and pharmacological manipulation (Chen et al., in press; Choy et al., 2016). In these systems, electrical activity can be recorded using different techniques such as monophasic action potential and bipolar electrogram methods, optical mapping and surface electrocardiography (Vigmond and Leon, 1999; van Rijen et al., 2001; Vigmond, 2005; Vigmond et al., 2009; Tse et al., 2016c,h). Although few mouse models have been generated specifically for studying IAB, experiments in different systems have increased our understanding on the molecular determinants of AP conduction (Tse et al., 2012, 2016d,e,i; George et al., 2015; Veeraraghavan et al., 2015) and how abnormalities in ion channels or cardiac remodeling lead to intra- or inter-atrial conduction defects (Figure 4).

Conduction between successive cardiomyocytes occurs via gap junctions. Each gap junction consists of two connexons, each of which is a hexamer of proteins called connexins (Cx). Cx40 and 43 are the isoforms expressed in the atria (Beyer et al., 1987; Gourdie et al., 1993a,b). Cx40^−∕−^ mice showed intra-atrial conduction delay (Hagendorff et al., 1999; Verheule et al., 1999). Mice with cardiomyocyte-directed expression of CREM-IbΔC-X, an isoform of transcription factor CREM (Kirchhof et al., 2013), showed evidence of fibrosis, atrial dilatation and IAB, associated with downregulation of Cx40 and ryanodine receptor 2 (RyR2)-mediated Ca^2+^ leak from the sarcoplasmic reticulum (Li et al., 2014). Increased leak could have a knock on effect by downregulating Na^+^ channels and decreasing *I*_Na_ (Curran and Louch, 2015).

Liver kinase B1, which has been termed the master upstream kinase, normally activates AMP-activated protein kinase (AMPK) and other related kinases (Gan and Li, 2014). Its deletion led to downregulation of both Cx40 and Na^+^ channels, resulting in complete absence of inter-atrial conduction (Kim et al., 2015). This was later complicated by atrial enlargement and fibrosis without inflammation, hypertrophy or apoptosis. Interestingly, mice with knockout of regulator of G-protein signaling 5 (Rgs5^−∕−^), a negative regulator of G protein-mediated signaling, showed increased P-wave duration in the absence of atrial dilatation or fibrosis (Qin et al., 2012). These two experiments on mouse model support the notion that although IAB and LAE frequently co-exist, they have different underlying pathologies.

### Increased oxidative stress, renin-angiotensin system activation and IAB

For cardio-metabolic disorders such as hypertension and diabetes, the common link appears to involve increased reactive oxygen species (ROS) production leading to excess oxidative stress (Tse et al., 2016a; Zhang et al., 2016). In hypertension, there is increased renin-angiotensin system (RAS) activation with elevated levels of angiotensin II (Murugan et al., 2015; Zhang et al., 2015). Mice infused with angiotensin II showed inter-atrial conduction delay, which was dependent on the leptin signaling pathway (Fukui et al., 2013). This resulted in upregulation of transforming growth factor beta, Monocyte Chemoattractant Protein-1 and RANTES, ultimately leading to fibrosis from deposition of collagen types 1 and 3. Ang-II has been shown to mediate cardiac fibrosis and inflammation via the Smad/NF-κB pathway (Wei et al., 2013). NF-κB can bind to the promoter region of the gene encoding for the Na^+^ channel (Shang and Dudley, 2005) to reduce its transcriptional activity (Shang et al., 2008).

Moreover, cardiac-restricted ACE overexpression produced conduction defects and reduced expression of atrial connexin 40 (Cx40) and connexin 43 (Cx43) proteins. Activation of the AT_1_ receptor by Ang-II stimulates Nox2 to generate oxygen free radicals, which can diffuse to and promote further ROS release at the mitochondria, by activation of the mitochondrial permeability transition (MPT) to mediate ROS-induced ROS release (RIRR; Zorov et al., 2000). Mitochondrial ROS can decrease the expression of Cx43 via c-src activation (Sovari et al., 2013), as well as induce myocardial fibrosis via NOX4/MAPK signaling (Aragno et al., 2006; Kuroda et al., 2010). Diabetes produces a cardiomyopathy characterized by diastolic dysfunction and structural remodeling. Cardiac fibrosis is observed in many models of diabetes. Thus, OVE26 mice modeling type 1 diabetes mellitus showed increased nuclear factor-κB and matrix metalloproteinase (MMP) activities and cardiac fibrosis (Li et al., 2011).

Leptin-deficient ob/ob mice modeling human diabetes mellitus showed reduced pro-MMP-8, -9, and -13 gene expression and increased stimulation of pro-collagen Iα, resulting in cardiac fibrosis (Zibadi et al., 2011). Leptin receptor-deficient db/db mice similarly show increased fibrosis (Cox and Marsh, 2014). In diabetes, RyR2 gating is abnormal due to channel oxidation by ROS (Eager et al., 1997; Xu et al., 1998; Bidasee et al., 2003) and phosphorylation by Ca^2+^/calmodulin-dependent protein kinase II (Witcher et al., 1991; Hain et al., 1995; Wehrens et al., 2004), which would lead to *I*_Na_ downregulation as discussed above. Taken together, inflammation or infiltration lead to electrophysiological remodeling of Na^+^ channel and gap junction downregulation, as well as structural remodeling of fibrosis. Together, these produce conduction abnormalities that underlie conduction block in Bayés syndrome.

## Future perspective

Our understanding of Bayés syndrome has increased significantly due to the development of mapping systems and the use of genetic and pharmacological mouse models for studying cardiac electrophysiology. Despite its clinical significance, this condition is under-diagnosed. Raising the awareness of IAB in healthcare professionals could improve its diagnostic rates (Baranchuk and Bayes de Luna, 2015). Risk stratification is important for determining individuals who are most at risk of cardiac arrhythmias (Tse, 2016a,b,c; Tse and Yan, 2016), and should include patients with Bayés syndrome (Martinez-Selles et al., 2016a). Measurement of magnetic fields in the heart has been useful for characterizing cardiac structural abnormalities (Vassiliou et al., 2014; Tse et al., 2015a,b), which can be useful for detecting atrial fibrosis. Magnetocardiography can be used to diagnose and predict the risk of cardiac arrhythmias in clinical practice (Steinhoff et al., 2004; Kuijpers et al., 2011; Sato et al., 2012; Kwong et al., 2013; Ito et al., 2014; Yoshida et al., 2015) and has the potential for early detection of IAB (Jurkko et al., 2009).

IAB results in delayed and asynchronous activation of the left atrium (Agarwal et al., 2003; Budeus et al., 2005; Caldwell et al., 2014). IAB, particularly in its advanced form, is frequently associated with supraventricular tachy-arrhythmias (Bayes de Luna et al., 1999; Conde et al., 2015) and higher cardiovascular and all-cause mortality (Ariyarajah et al., 2007c; Magnani et al., 2011). The pathogenesis of AF in the context of IAB has been studied in detail, demonstrating the occurrence of the following event sequence: abnormal atrial activation can lead to increased atrial pressure, with subsequent electrophysiological and structural remodeling, such as atrial dilatation and fibrosis. Furthermore, endothelial damage and dysfunction, together with impaired atrial mechanical activity, is thrombogenic (Martinez-Selles et al., 2016a). Regarding the optimal management of IAB and AF, anti-arrhythmic treatment can reduce the recurrence rate of atrial fibrillation (AF) associated with IAB (Bayes de Luna et al., 1988, 1989). Anti-coagulation is needed to reduce the risk of thrombo-embolic complications. Anti-coagulation is likely to be beneficial for patients with IAB, even before the development of AF (Martinez-Selles et al., 2016a). A recent paper proposed that anticoagulation treatment should be initiated based on the following criteria: P wave duration ≥ 160 ms, structural heart disease, >40 atrial premature beats/h and/or runs in Holter monitoring and CHA2DS2-VASc score ≥ 2. ACE inhibitors are now increasingly recognized for their anti-fibrotic effects and trials should be conducted to determine their relative efficacies in reducing arrhythmic risk, morbidity and mortality in patients with IAB. Interventional management, such as synchronous biatrial pacing, can be used to prevent the recurrences of AF associated with IAB (D'Allonnes et al., 2000). However, resynchronization therapy may be difficult in situations such as hypertrophic cardiomyopathy or heart failure, where patients will have abnormal cardiac hemodynamics. Thus, future studies will be needed for its clarification.

## Author contributions

GT: Design of manuscript; drafted and critically revised the manuscript for important intellectual content; preparation of figures. EL: Acquired and interpreted primary research papers; critically revised the manuscript for important intellectual content; preparation of figures. JY: Analyzed and interpreted primary research papers; critically revised the manuscript for important intellectual content. BY: drafted and critically revised the manuscript for important intellectual content. All authors approved the final version, ensured that the text is accurate and agreed to be accountable for all aspects of the work.

### Conflict of interest statement

The authors declare that the research was conducted in the absence of any commercial or financial relationships that could be construed as a potential conflict of interest.
